# Narrative Review: Submental Artery Island Pedicled Flap, Indications, Tips, and Pitfalls

**DOI:** 10.1177/19433875231208565

**Published:** 2023-10-13

**Authors:** Camilo Mosquera, Carlos Ramirez

**Affiliations:** 1 Division of Oral and Maxillofacial Surgery, Department of Surgery, 12338 University of Texas Medical Branch, Galveston, TX, USA; 2 Department of Oral and Maxillofacial Surgery, Head and Neck Oncologic Surgery and Microvascular Reconstruction, 21927 Ascension Macomb-Oakland Hospital, Detroit, MI, USA

**Keywords:** island flap, pedicled flap, myocutaneous flap, submental flap, reconstructive surgical procedures, head and neck neoplasm

## Abstract

**Study Design:**

Narrative review.

**Objective:**

To describe the advantages, challenges, and potential indications of the submental artery island flap as a local pedicled flap for head and neck reconstruction.

**Methods:**

We conducted a comprehensive review of the literature to describe the submental artery island flap's surgical technique, indications, and outcomes. Data sources included peer-reviewed articles, case reports, and clinical studies on using the submental flap in head and neck surgery reconstruction.

**Results:**

The submental artery island flap, while offering advantages such as minimal donor site morbidity, and good cosmetic outcomes, presents challenges related to the pedicle dissection and patient selection. This flap is particularly suitable for defects in the oral cavity, oropharynx, parotid bed, and midface or neck skin. Simultaneous neck dissection is feasible but should be approached with caution in patients with a history of prior neck surgery.

**Conclusions:**

The submental artery island flap is a valuable option for selected cases. When performed in the right patient with a correct technique, this flap can reconstruct defects in the oral cavity, oropharynx, parotid bed, and skin of the midface or neck with a minimally visible scar of the donor site.

## Introduction

The submental artery island pedicled flap was initially introduced by Martin et al in 1993.^1^ It was described as a reliable alternative to the free flaps for head and neck reconstruction after oncologic procedures.^2^ It has been described for defects of the tongue, floor of the mouth, buccal mucosa, palate, oropharynx, hypopharynx, lateral skull base, and lower and mid face. When used for the reconstruction of skin defects, it matches the recipient site in terms of color and texture, and the donor site scar is well hidden, especially when the patient is standing upright.

Despite the reliability of the submental flap, it has struggled to gain a strong foothold due to the increased difficulty of dissection of the flap along the submandibular gland area and concerns about its use in patients with oral cavity cancers due to the belief of potentially transferring nodal disease to the reconstructed site; however, existing literature has provided evidence supporting the oncologic safety on this technique.^3,4^

### Anatomy

The submental artery island flap is a type C fasciocutaneous flap based on the submental artery, branch of the facial artery.^5^ The submental artery starts at 27.5 mm from the facial artery origin, 5.0 mm from the mandibular border, and 23.8 mm from the mandibular angle. The average diameter of the submental artery is 1.7 mm at the origin.^5,6^ After originating from the facial artery, the submental artery gives branches to the submandibular gland, the platysma, digastric and mylohyoid muscles, small branches to subplatysmal fatty tissue, and 1–4 cutaneous perforators.^7^ The submental artery can terminate superficial to, or within the anterior belly of the digastric muscle. During flap harvest, the submental artery has been found to lie deep to the muscle in 70% of the cases.^8^ The pedicle length ranges from 5 to 8 cm. The venous drainage is the submental vein that drains to the facial vein with a mean diameter of 2.2 mm.

The submental artery has been reported to be superficial to the submandibular gland in 69% of the cases, and in one case, it was found to be passing through the gland. This is an anatomical variation that the reconstructive surgeon must be aware of since it can significantly increase the complexity of the dissection of the pedicle.^6^ Additionally, it has been reported originating directly from the carotid artery in one case,^1^ and the marginal mandibular nerve has been found adjacent to the artery, which should also be considered during the operation to prevent nerve deficits.^6^

### Indications and Goals

This flap is a reliable option for reconstructing small to significant defects in the head and neck with still primary closure of the donor site. The pedicle length gives a substantial arc of rotation extending from the medial canthus to the zygomatic arc. The flap is an alternative to free flaps in patients who are not candidates for free tissue transfer. The location of the donor site scar is well hidden and barely noticeable Table 1.

**Table 1.** Summary of the Indications, Contraindications, Advantages, and Disadvantages of Using the Submental Flap.

Aspect	Submental flap
Indications	Small to moderate-size defects
	• Patients not candidates for free tissue transfer
	• Defects in oropharynx, maxilla, tongue, floor of the mouth, buccal mucosa, palate, and lower and mid-face
Contraindications	• Unsuitable vascular supply in submental region
	• History of prior neck dissection^a^
	• Extensive neck involvement^b^
	• Inadequate skin paddle size for defect
	• Patients with unfavorable body habitus
Advantages	• Minimal donor site morbidity
	• Hidden donor site scar
	• Primary closure of donor site
	• Good color and texture match for skin defects
	• Can be raised simultaneously with neck dissection
Disadvantages	• Risk of vascular compromise with neck dissection
	• Careful patient selection required
	• Potential for limited skin paddle size
	• Inadequate for extensive or complex defects
	• Challenging dissection of pedicle for unexperienced surgeons

^a^Relative contraindication.

^b^In cases where there is suspicion of skin involvement.

In this review, we aim to outline submental artery island pedicled flap reconstruction from the preoperative to postoperative stages, highlighting tips and pitfalls experienced by our team.

## Material and Methods

### Preoperative Preparation

Preoperative evaluation of the neck is crucial to determine if the patient is a good candidate for submental artery pedicled flap reconstruction and should include assessment for previous surgeries or trauma in the submental area. There are few reports in the literature reporting the success of the submental flap in patients with history of neck dissection; the authors often perform the flap simultaneously with ispilateral neck dissection. However, when there is history of contralateral neck dissection, the possibility of the previous sacrifice of the submental artery can jeopardize the outcome of the flap.^9^ A detailed review of the previous operative notes is advised to identify potential signs suggesting this. Larson et al.^9^ reported a small series of cases in which a submental flap was used when a contralateral neck dissection had previously been performed with excellent outcomes without evidence of partial or total flap loss. A doppler scan can be used to trace the trajectory of the submental artery and identify the maintenance of the submental perforator in the neck opposite to the previous neck dissection.^9^

The size of the needed skin paddle is measured; this is done with a pinch test of the skin inferior to the inferior border of the mandible and will tell if the remaining skin from the neck can be advanced for primary closure. The other important information needed is the location of the defect. This flap can reach oral cavity, oropharynx, hypopharynx, maxilla, and various sites in the face, such as the parotid bed, chin, face, upper and lower lip, and neck defects.

### Procedure

**Figure 1.**
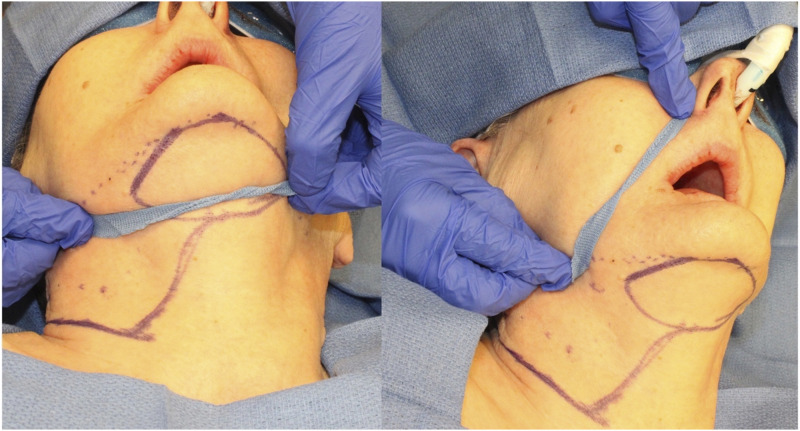
Measurement of the arc of rotation and design of skin incision to perform neck dissection and thyroid lobectomy simultaneously.

**Figure 2.**
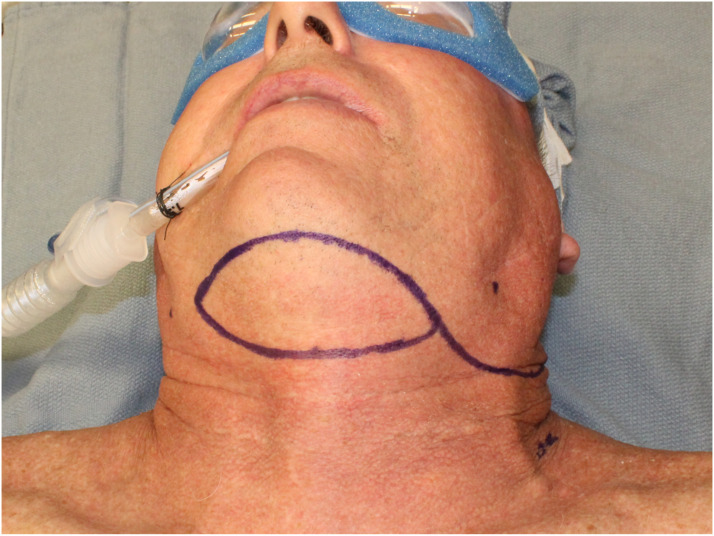
Design of skin paddle.

The patient is positioned supine with extension of the neck using a shoulder roll and breaking the head of the surgical bed. Using either a suture string or a string from the lap towel, one end is positioned on the ipsilateral side of the defect on the submandibular area inferior to the antegonial notch and pivoted lying in a passive form into the farthest point of the defect. The string is then rotated caudally to the submental area and the lateral most reach is marked; this will be the most lateral portion of the skin paddle (Figure 1). Once the amount of skin is determined, an elliptical island is marked out as needed; this can extend laterally to the contralateral posterior border of the mandible or the mastoid area, if the flap is being raised in conjunction with neck dissection, the ipsilateral posterior border of the skin island is extended inferior and posterior to continue with the incision for the neck dissection (Figure 2).

The incision is extended from the skin, the subcutaneous tissue, and to the fascia on the contralateral anterior belly of the digastric; the incision is also extended inferiorly towards the ipsilateral side of the pedicle. Next, subplatysmal dissection starting on the contralateral side is done towards the midline down to the mylohyoid muscle; at this point, dissection continues towards the medial border of the ipsilateral anterior belly of the digastric.

The ipsilateral attachments of the ipsilateral anterior belly of digastric to the muscle and the tendon are divided and the muscle is sutured loosely to the skin paddle to prevent shearing of the perforators; at this point, the vascular pedicle supplying the skin can be identified running under the anterior belly of the digastric, care must be taken to prevent section of the submental artery inadvertently during the remaining dissection.

At this point, dissection is continued towards the facial artery, hugging the inferior border of the mandible following the submental artery and vein that runs horizontally.

**Figure 3.**
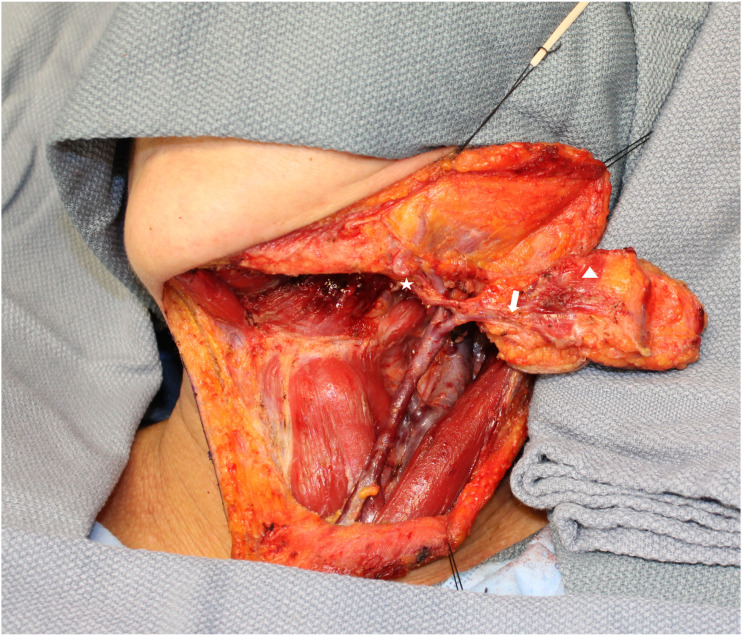
Flap raised and submental artery traced to origin at facial artery. (*Star: Facial Artery, *Arrow: Submental artery incorporating into the flap, *Arrowhead: Ipsilateral anterior belly of digastric muscle incorporated into flap).

Next, attention will be turned to the ipsilateral submandibular gland excision, subplatysmal flaps are elevated, and the fascia over the submandibular gland is raised to protect the marginal branch of the facial nerve. The submental artery is traced back to its origin at the facial artery with a careful dissection through the submandibular gland which can be excised at this point to increase visibility; at this point, the submental artery and vein will be dissected entirely from its takeoff point at the facial artery, and the rotation of the skin paddle can be verified (Figure 3).

Next, the flap is usually transferred to the recipient site by tunneling from the neck. After the flap is inset in the recipient site, the neck is closed over a suction drain, excess skin is excised, and the scar is hidden under the inferior border of the mandible.

### Tongue Reconstruction

**Figure 4.**
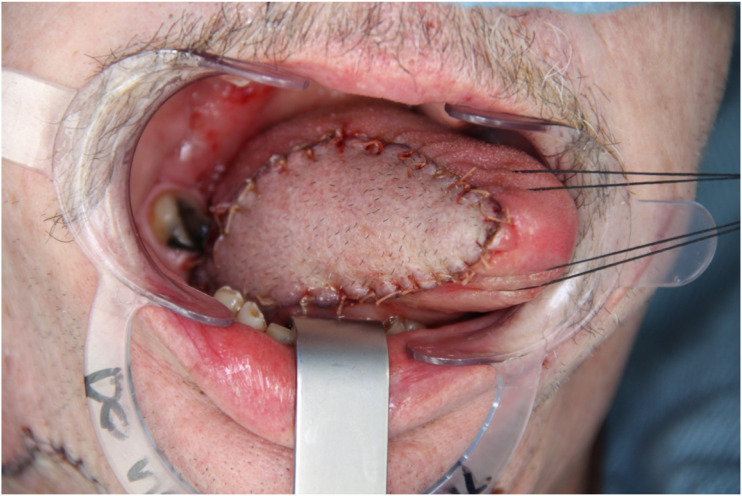
Tongue reconstruction with submental flap.

Small and medium size lateral tongue defects up to hemiglossectomy can be reconstructed with the submental artery island pedicled flap; a tunnel must be created between the mylohyoid muscle and the medial cortex of the mandible to transfer the flap to the oral cavity. Care must be taken to prevent constriction of the pedicle at this level which can compromise the venous outflow of the skin paddle (Figure 4).

### Mandible Reconstruction

**Figure 5.**
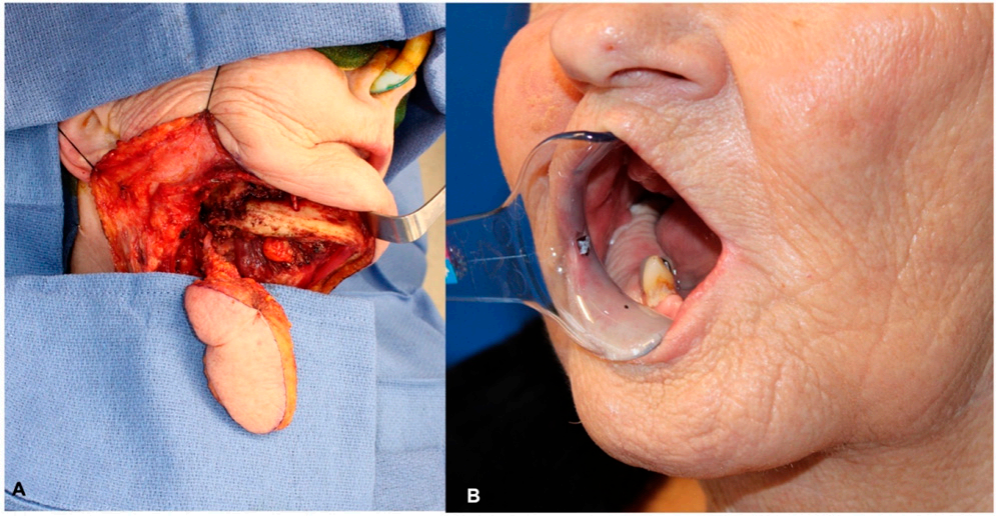
(A) Submental flap raised for mandibular reconstruction. (B) Late post-op with skin paddle reconstructing mandibular alveolar mucosa.

Posterior, lateral, and anterior marginal mandibulectomy defects can be nicely reconstructed with the submental flap by transferring it to the oral cavity medially to the mandible and then rotating the skin paddle buccally over the remaining mandible; this will create a smooth neo-buccal vestibule and will avoid formation of a bulky cheek; as it was transferred lateral to the mandible, care must be taken not to over transfer the skin paddle buccally to prevent the creation of a deep buccal vestibule that can be difficult to clean by the patient (Figure 5).

### Maxilla Reconstruction

Maxilla reconstruction is one of the best uses of the submental flap; the amount of tissue harvested from the submental area can easily reconstruct hemimaxillectomy defects with minimal donor site morbidity.

**Figure 6.**
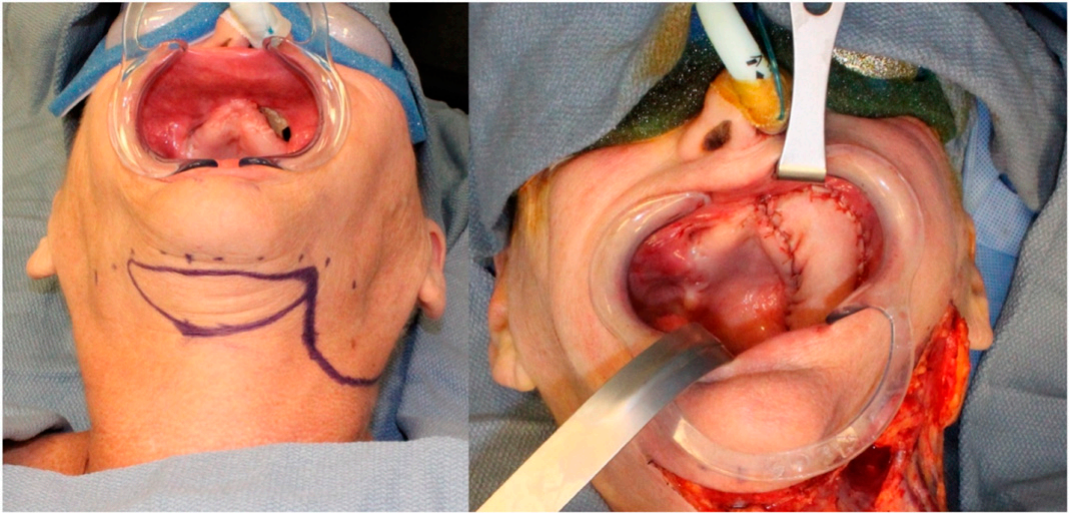
Maxillary reconstruction.

The flap is transferred to the oral cavity in the same fashion via a tunnel medially to the mandible through the mylohyoid muscle in the posterior aspect of the oral cavity; if further mobilization of the flap is required, this can be achieved by ligating and dividing the facial artery proximally over the mandibular body (Figure 6).

### Midface Reconstruction

**Figure 7.**
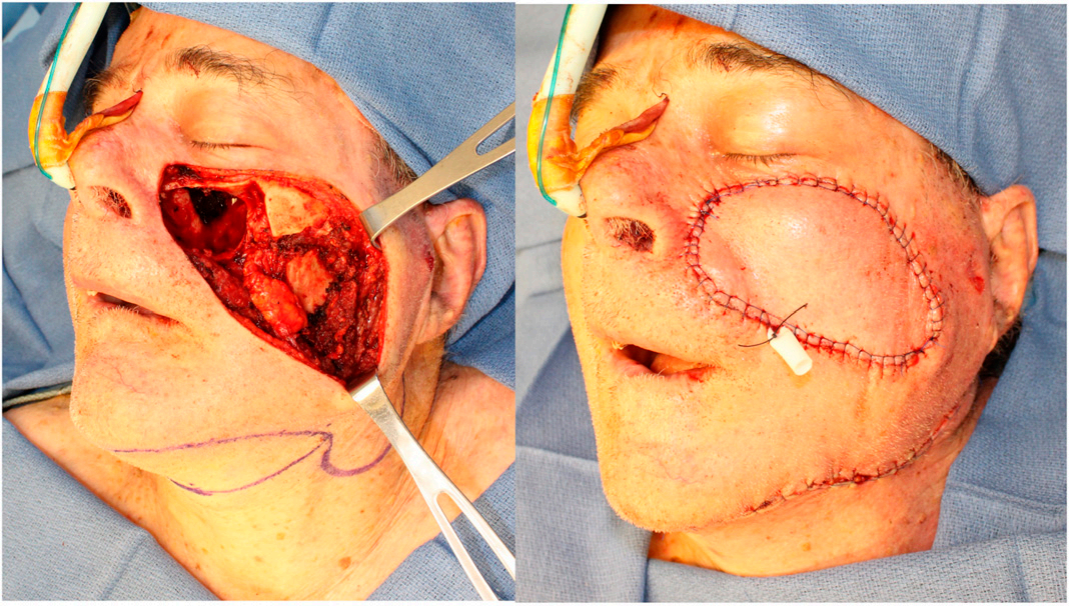
Midface reconstruction.

The great versatility and arc of rotation of the submental flap make it an excellent option for the reconstruction of soft tissue defects in the midface; a tunnel can be made on the subfascial plane, and the flap can be transferred to the midface; once again, division of the proximal facial artery over the mandibular body might be needed to increase the arc of rotation of the flap. The skin of the submental area is usually very pliable and provides excellent color match (Figure 7).

### Oncologic Safety

Some retrospective studies have assessed the oncologic safety of the submental flap due to false belief or fear of potentially transferring nodal disease to the reconstruction site.^3,4,10,11^ This concern has not been validated by the literature or the collective experience of most surgeons who use this flap routinely. The oncologic and reconstructive surgeon must be comfortable preserving the submental vasculature while dissecting levels 1A and 1B.

Elzahaby et al reported a cohort of thirty-six consecutive patients with oral squamous cell carcinoma who underwent reconstruction with submental island flap and simultaneous ipsilateral selective neck dissection of levels I–IV. In their cohort, none of the patients developed regional nodal recurrence, and three patients developed local recurrence that was treated with re-resection.^12^

When performed simultaneously with neck dissection, the authors prefer to raise the flap early at the beginning of the case before proceeding with the neck dissection; this allows them to assess the viability of the flap after dissecting the different levels of the neck. For the neck dissection, the authors use bipolar electrocautery, which is precise and allows them to carefully dissect lymph nodes in level 1, reducing the chances of unintentionally harming the blood supply to the submental flap. This approach ensures that the submental flap remains viable, and that level 1 is properly dissected, serving both reconstructive and oncologic goals effectively.

Although the submental flap is very reliable in expert hands, it is recommended to have a backup reconstruction plan in case the submental pedicle is injured during the neck dissection.

### Postoperative Care

After the submental island flap, patients are transferred to a stepdown unit if there’s any concern of acute postoperative respiratory failure for airway surveillance or to a regular floor. Patients initiate clear liquids diet 24 h hours after the procedure and transition to a full liquid diet upon discharge until 1-week post-op when is advanced to soft diet as tolerated; the suction drain is kept in place until the output is under 25 mL over 24 h, and patients are educated on restricted neck mobility for the first week to reduce risk of neck wound dehiscence.

### Complications

**Figure 8.**
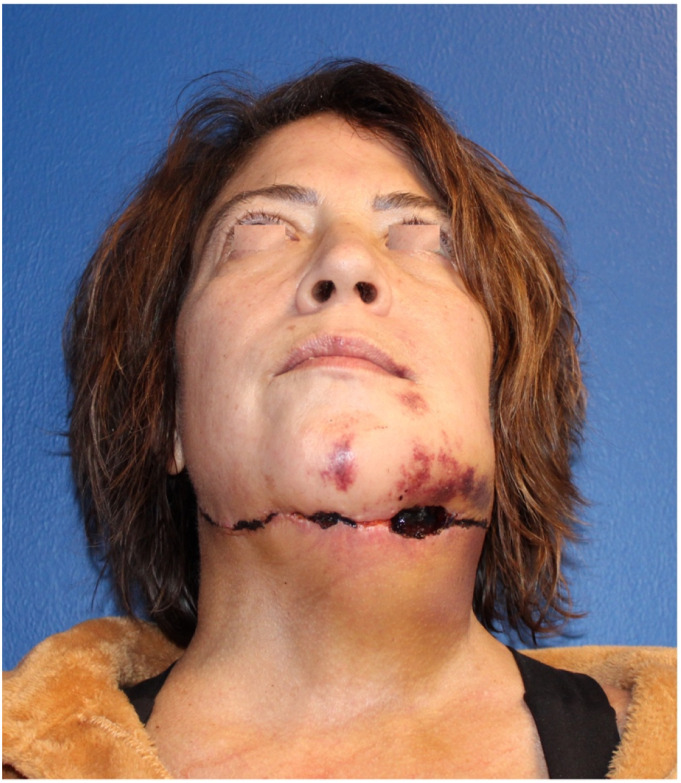
Wound dehiscence secondary to hematoma after submental flap.

Partial or total flap failure is the most concerning complication that can be associated to the submental island pedicled flap; this could be related to injury to the vascular pedicle during the dissection. Other potential complications associated with the harvesting of the flap are dehiscence of the donor site, postoperative hematoma (Figure 8), and injury to the marginal mandibular branch of the facial nerve. Literature has shown that perioperative complications between the submental flap and free tissue transfer are mostly comparable; however, the submental flap can present a higher rate of partial flap necrosis.^13^

## Discussion

### Tips and Pitfalls

#### Preoperative

(I) A pinch test must be performed to assess the amount of skin that can be harvested while allowing primary closure of the donor site.
(II) Superior incision must be designed following the inferior border of the mandible to have better aesthetic results.
(III) History of neck dissection must be assessed since the possibility of the previous sacrifice of the submental artery can jeopardize the viability of the flap.

#### Procedure

(I) The ipsilateral anterior belly of the digastric should be incorporated into the flap and sutured to the skin paddle to prevent inadvertent shearing of the small perforators.
(II) After identifying the vascular pedicle at the level of the ipsilateral digastric, dissection of the submental artery towards the facial artery must be done; at this point, careful dissection with bipolar must be done through the submandibular gland to prevent injury of the vascular pedicle.
(III) Transfer of the skin paddle to the oral cavity should be done medially to the mandible through the mylohyoid muscle to prevent the formation of bulkiness in the lateral aspect of the jaw; the mandible will also serve as protection to the vascular pedicle.

#### Postoperative

**Figure 9.**
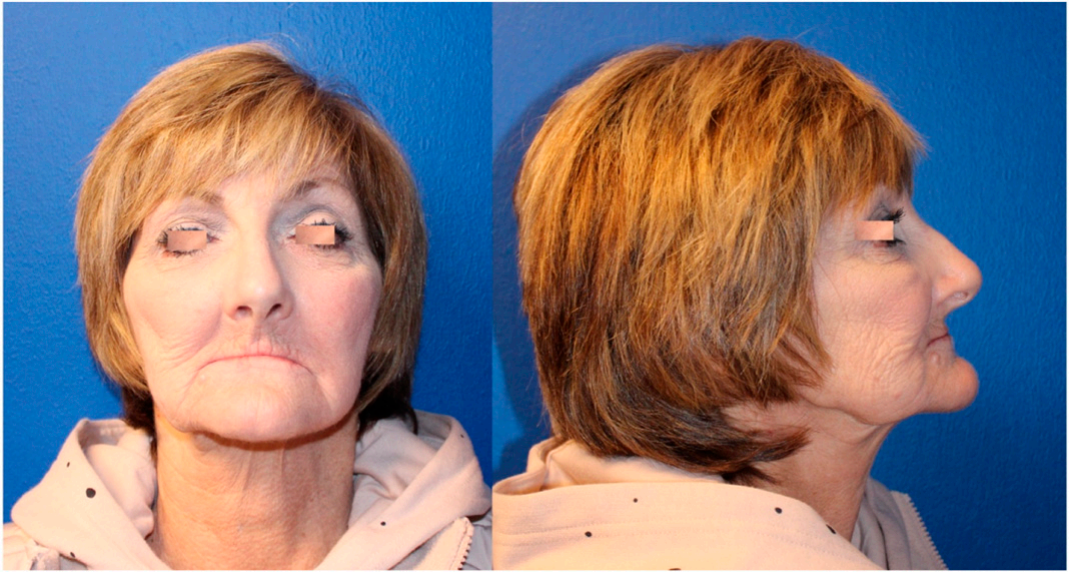
Late post-op with minimal scar in donor site.

(I) A suction drain must be kept in place to eliminate the dead space and prevent formation of seromas or hematomas.
(II) Patients must be instructed on restricted neck mobility for the first week post-op to prevent wound dehiscence (Figure 8).
(III) Standard wound care in late postop will ensure adequate healing of the donor site with minimally visible scar (Figure 9).

The submental artery island flap is a versatile head and neck reconstruction option with minimal donor site morbidity allowing primary closure. By having a good arc of rotation that can be extended, this flap can easily reach defects in the oral cavity, oropharynx, parotid bed, and skin of midface and neck with a good color match. This flap offers several advantages compared to other flaps than can be used to reconstruct these areas; the location of the donor site in the submental region results in minimal visible scarring, and patients often appreciate the aesthetic outcome. The submental flap can be harvested simultaneously with neck dissection for oncologic cases while allowing a comprehensive dissection of level 1 of the neck without compromising the oncologic principles.

While the submental flap offers these advantages, it’s essential to recognize that no single flap is universally superior in all situations. Other regional or free flaps may be preferred in specific cases, based on factors like defect size, location, and patient characteristics. Therefore, the choice of flap should be tailored to the individual patient’s need and the clinical context to achieve the best possible outcome.
